# Peroperative administration of tranexamic acid in sleeve gastrectomy to reduce hemorrhage: a double-blind randomized controlled trial

**DOI:** 10.1007/s00464-023-10232-5

**Published:** 2023-07-03

**Authors:** J. W. H. ‘t Hart, B. J. Noordman, J. M. A. Wijnand, L. U. Biter, S. J. C. Verbrugge, E. Birnie, M. Dunkelgrun, J. Huisbrink, J. A. Apers

**Affiliations:** 1 Department of Surgery, Franciscus Gasthuis & Vlietland, Kleiweg 500, 3045 PM Rotterdam, The Netherlands; 2 Department of Pharmacology, Franciscus Gasthuis & Vlietland, Rotterdam, The Netherlands; 3 Department of Anesthesiology, Franciscus Gasthuis & Vlietland, Rotterdam, The Netherlands; 4 Department of Statistics and Education, Franciscus Gasthuis & Vlietland, Rotterdam, The Netherlands; 5 Department of Surgery, Tulp Medisch Centrum, Zwijndrecht, The Netherlands; 6 Department of Surgery, Erasmus University Medical Centre, Doctor Molewaterplein 40, 3015 GD Rotterdam, The Netherlands; 7 Department of Genetics, University Medical Center Groningen, University of Groningen, Hanzeplein 1, 9713 GZ Groningen, The Netherlands

**Keywords:** Tranexamic acid, Hemorrhage, Fast-track, Hemostatic clips, Laparoscopic sleeve gastrectomy

## Abstract

**Introduction:**

In metabolic surgery, hemorrhage is the most common major complication. This study investigated whether peroperative administration of tranexamic acid (TXA) reduced the risk of hemorrhage in patients undergoing laparoscopic sleeve gastrectomy (SG).

**Methods:**

In this double-blind randomized controlled trial, patients undergoing primary SG in a high-volume bariatric hospital were randomized (1:1) to receive 1500-mg TXA or placebo peroperatively. Primary outcome measure was peroperative staple line reinforcement using hemostatic clips. Secondary outcome measures were peroperative fibrin sealant use and blood loss, postoperative hemoglobin, heart rate, pain, major and minor complications, length of hospital stay (LOS), side effects of TXA (i.e., venous thrombotic event (VTE)) and mortality.

**Results:**

In total, 101 patients were analyzed and received TXA (*n* = 49) or placebo (*n* = 52). There was no statistically significant difference in hemostatic clip devices used in both groups (69% versus 83%, *p* = 0.161). TXA administration showed significant positive changes in hemoglobin levels (millimoles per Liter; 0.55 versus 0.80, *p* = 0.013), in heart rate (beats per minute; -4.6 versus 2.5; *p* = 0.013), in minor complications (Clavien–Dindo ≤ 2, 2.0% versus 17.3%, *p* = 0.016), and in mean LOS (hours; 30.8 versus 36.7, p = 0.013). One patient in the placebo-group underwent radiological intervention for postoperative hemorrhage. No VTE or mortality was reported.

**Conclusion:**

This study did not demonstrate a statistically significant difference in use of hemostatic clip devices and major complications after peroperative administration of TXA. However, TXA seems to have positive effects on clinical parameters, minor complications, and LOS in patients undergoing SG, without increasing the risk of VTE. Larger studies are needed to investigate the effect of TXA on postoperative major complications.

**Graphical abstract:**

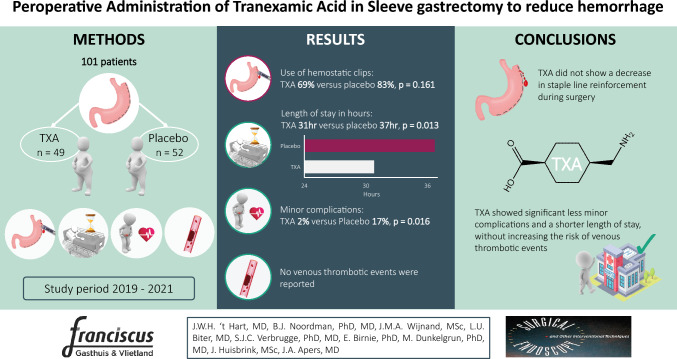

**Supplementary Information:**

The online version contains supplementary material available at 10.1007/s00464-023-10232-5.

The prevalence of extreme obesity is rising, more metabolic procedures are performed, and more patients are exposed to the risks of peroperative and postoperative complications [1]. In metabolic surgery, prevention of venous thrombotic events (VTE) and prevention of hemorrhage are both challenges to be encountered [2, 3]. Postoperative hemorrhage occurs in 2.0–4.0% of patients undergoing laparoscopic sleeve gastrectomy (SG) [4–9]. Fast-track metabolic surgery is safe and can lower the risk of VTE, due to early mobilization of the patients [10, 11]. However, when patients are discharged early after surgery, even more in day-care setting, the clinical window to detect hemorrhages is narrow. Therefore, fast track metabolic surgery warrants reduction of hemorrhage. Several factors to prevent hemorrhage, such as staple height, stapler closure time before gastric dissecting, and different techniques of staple line reinforcement (SLR), have been investigated [12]. However, none of these factors significantly reduced the incidence of hemorrhage [7, 13, 14].

In other types of surgery, such as cardiothoracic and orthopedic surgery, tranexamic acid (TXA) has been proven to be effective in reducing hemorrhage. In these studies, administration of TXA was safe, as no increase in VTE was seen in these patients [15–17]. In metabolic surgery, the effectiveness of TXA has not yet been investigated thoroughly. In a non-randomized study described by Chakravartty et al., less bleeding points and a shorter operation time were seen in 25 patients who received TXA, compared to 25 patients in the control group. The authors concluded that administrating TXA was a simple and economically effective option, as TXA is an inexpensive drug [18]. Klaassen et al. evaluated the benefit of postoperative administration of TXA in 44 patients with hemorrhagic complications. They saw no VTE and no reoperations were required in 40 patients [8].

The effectiveness of TXA in metabolic surgery has not been investigated in a randomized controlled trial. The aim of the current trial was to investigate if peroperative administration of TXA could reduce the per- and postoperative hemorrhage rates in patients undergoing SG, without increasing the risk of VTE.

## Methods

### Design and patients

The study protocol has been published previously (supplementary file 1) [19]. In brief, we conducted a double-blind randomized controlled trial in a high-volume bariatric hospital. At the outpatient clinic, the coordinating researcher recruited all patients with sufficient Dutch or English language proficiency who were planned to undergo primary SG and met the international guidelines for metabolic surgery [20]. Exclusion criteria were use of anticoagulants, medical history of VTE (defined as deep vein thrombosis or pulmonary embolism) or hemorrhage, and arterial and/or severe iatrogenic bleeding during surgery. Enrollment stopped in September 2021 when the inclusion target for the study was achieved. This study was registered with the Netherlands Trial Register (NL8029).

### Randomization and masking

To give the pharmacologist sufficient time to prepare the infusion bags, patients were randomly allocated (1:1) one week before surgery by the coordinating researcher via computer-generated variable block randomization software by Ciwit BV (Castor EDC©). The patient, surgical team, and anesthesia team were blinded for treatment allocation. The coordinating researcher and hospital pharmacologist were unblinded to properly prepare the infusion bags for each individual patient. Both were not involved in the surgical procedure nor in the follow-up.

### Procedures

The intervention group received a single dose of 1500-mg TXA (Cyklokapron©) during the induction of the procedure. TXA was administered intravenously, dissolved in 100-ml sodium chloride (NaCl) 0.9% in a time frame of 15–30 min, with a maximum of 100 mg/min. The control group received a placebo during the induction of the procedure. The placebo infusion contained 100-ml NaCl 0.9%, which was administered similarly. The hospital’s pharmacy prepared and labeled the investigational medicinal products according to the relevant Good Manufacturing Practice (GMP) guidelines [21].

To determine postoperative hemoglobin levels, a blood sample (one EDTA tube) was obtained within 1 week preoperatively and at day one postoperatively by venipuncture and analyzed in the hospital’s clinical chemistry laboratory according to standard procedures. In all patients, the same staple devices (ECHELON FLEX™ GST) to dissect the stomach and energy device (Harmonic®) were used, starting always with a golden cartridge (3.0 mm), followed by blue (2.4 mm) and rarely green (3.4 mm). All patients were treated by expert bariatric surgeons. Staple line hemorrhage was checked according to a step-by-step protocol (see supplementary files 1 and 2 [19]). After the greater curvature of the stomach was removed from the abdomen, the patient’s blood pressure was brought back to normotension, and the abdominal pressure was lowered to 12 mmHg. The staple line was inspected when normotension was reached. If blood pumped out of the staple line (active bleeding), hemostatic clips (Ethicon Ligaclip®) were applied. If blood was only oozing (passive bleeding), fibrin sealant was applied. Patients with iatrogenic bleeding from another location than the staple line were excluded from the study. Postoperatively, patients received intravenous administration of NaCl 0.9% (≤ 500 ml per 24 h) and 5000 units (prophylactic) low-molecular weight heparin (LMWH) at 10 PM. If patients were hospitalized longer, they continued daily prophylactic LMWH. Patients never received LMWH preoperatively, as is common in Dutch hospitals [10, 22]. All primary and secondary outcomes were prospectively registered in digital case report forms (Castor EDC). Vital signs were measured at 6 AM before mobilization. When postoperative hemoglobin levels were decreased ≥ 1.5 points or ≥ 1.0 point in the presence of clinical symptoms of hemorrhage (such as lightheaded, fainting, tachycardia), the bariatric surgeon was consulted, additional TXA was prescribed, and extra hemoglobin monitoring was ordered. The abdomen was inspected at day one and one week after surgery for abdominal wall hematoma. If there was a high suspicion of a hemorrhage postoperatively, a CT scan was performed. Adverse events were reported to the medical research ethics committee and the national trial committee. All patients underwent SG as described previously [23].

### Outcomes

The primary outcome measure was peroperative hemorrhage defined as SLR using hemostatic clips. Secondary outcome measures were peroperative use of fibrin sealant, blood loss, length of procedure (LOP), postoperative difference in hemoglobin (millimole per liter (mmol/L)), heart rate (beats per minute (bpm)), pain, extra hemoglobin monitoring, major complications due to hemorrhage (scored as Clavien–Dindo ≥ 3, i.e., needing packed red blood cells or surgical/radiological intervention ≤ 30 days postoperatively), minor complications due to hemorrhage (scored as Clavien–Dindo ≤ 2; i.e., extra TXA administration, hematemesis/melena, infected abdominal wall hematoma), length of hospital stay (LOS), general complications, side effects of TXA (VTE, hypotension, nausea and vomiting), and mortality within 3 months postoperative [24].

### Statistical analysis

The estimated sample size was 2 × 50 = 100 patients. The analysis was based on an expected 50% decrease of the percentage of patients for whom peroperative placement of hemostatic clips was required (power = 80%, *α* = 5% two-sided) and an expected exclusion rate of 28%.

Statistical analyses were performed using IBM SPSS version 28 (IBM Corporation, Armonk, New York, USA). Outcomes were described as absolute number with percentage for categorical variables and as mean with standard deviation (SD) for parameters with normal distribution. For skewed data we used log transformation to create normally distributed data.

Differences in categorical data were tested using the Chi-squared test or Fisher’s exact test and differences in continuous data were tested using the independent sample t test or Mann–Whitney U test, as appropriate. In addition to the primary statistical tests, exploratory analyses, including Chi-squared test and linear or logistic regression analysis, were conducted to assess potential associations between variables of interest. Results were evaluated at a significance threshold of *p* < 0.05 (two-sided). Data were analyzed according to the as-treated principle.

## Results

**Fig. 1:**
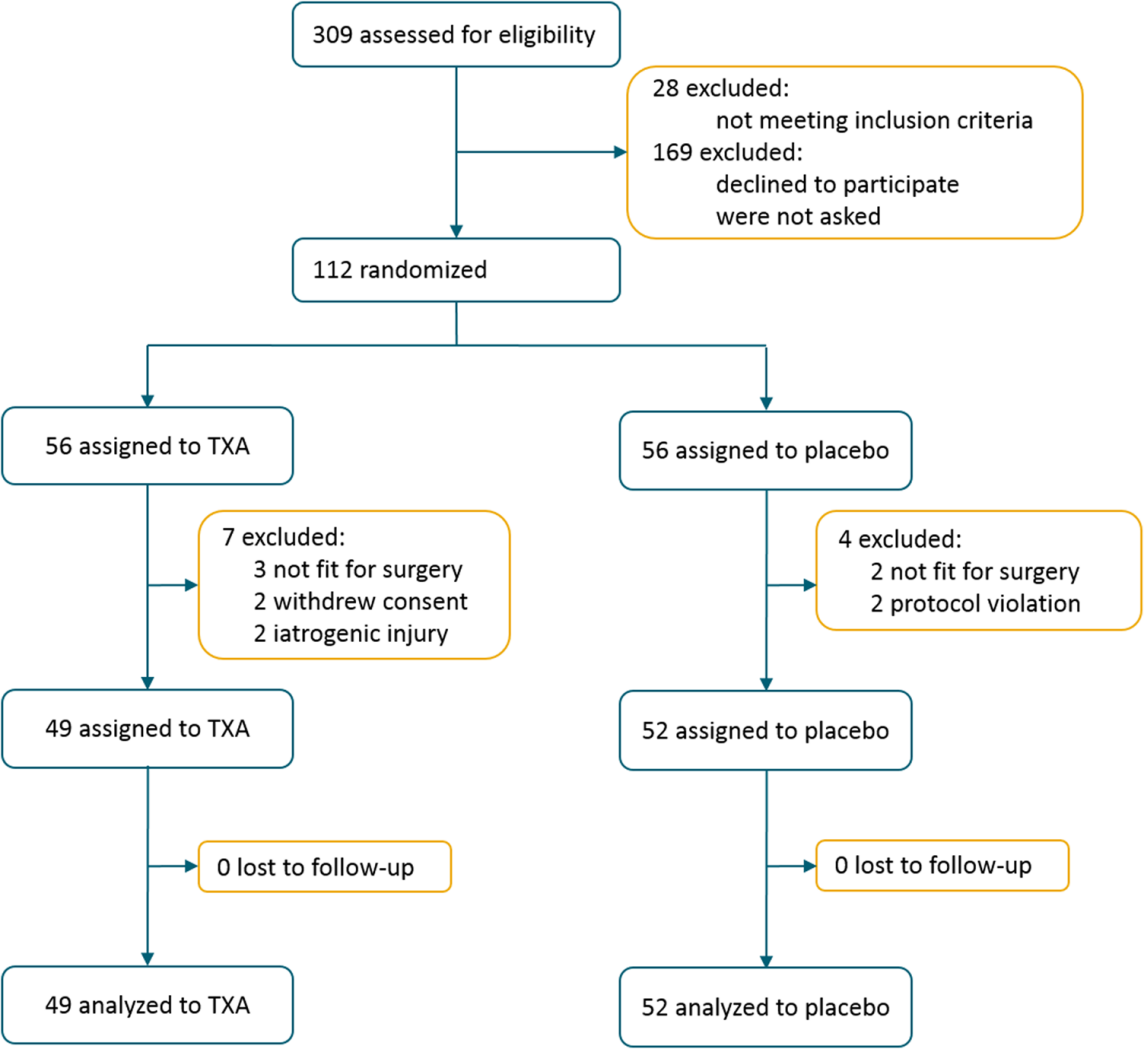
Flowchart. *TXA* tranexamic acid

Between July 2, 2020, and August 23, 2021, 309 patients were assessed for eligibility. In total, 112 eligible patients were randomized to receive either TXA or placebo (Fig. 1). Eleven patients were excluded (five patients were deemed not fit for surgery by the anesthesiologist, two had peroperative iatrogenic arterial injury, two patients withdrew informed consent, and in two patients there was protocol violation). Between September 1, 2020 and October 12, 2021, 101 patients received the allocated treatment, 49 patients TXA and 52 patients placebo. All 101 patients completed 3-month follow-up. Baseline characteristics were comparable between both groups (Table 1).

**Table 1:** Baseline characteristics

	TXA (*n* = 49)	Placebo (*n* = 52)	*p*-value
Age (years) (*mean, SD*)	36.0 (10.9)	36.8 (12.3)	*p* = 0.632^a^
Weight (kg) (*mean, SD*)	118.8 (19.8)	120.1 (24.4)	*p* = 0.671^a^
Body Mass Index (kg/m2) (*mean, SD*)	42.3 (5.4)	41.5 (5.3)	*p* = 0.444^a^
Female gender	38 (77.6%)	42 (80.8%)	*p* = 0.690^b^
Hypertension	8 (16.3%)	12 (23.1%)	*p* = 0.395^b^
Diabetes mellitus	2 (4.1%)	3 (5.8%)	*p* = 1.000^c^
Dyslipidemia	5 (10.2%)	5 (9.6%)	*p* = 1.000^c^
Obstructive sleep apnea	17 (34.7%)	18 (34.6)	*p* = 0.993^b^
Severe joint pain	8 (16.3%)	8 (15.4%)	*p* = 0.897^b^
Gastroesophageal reflux disease	4 (8.2%)	5 (9.6%)	*p* = 1.000^c^
Possible interacting medication (risk > 0.1%)	6 (12.2%)	4 (7.7%)	*p* = 0.518^c^
Heart rate (bpm) (*mean, SD*)	81.8 (10.3)	79.2 (12.4)	*p* = 0.245^a^
Systolic blood pressure (mmHg) (*mean, SD*)	129 (13.4)	132 (16.9)	*p* = 0.254^a^
Diastolic blood pressure (mmHg) (*mean, SD*)	75 (10.7)	74 (12.1)	*p* = 0.599^a^
Hemoglobin (mmol/L) (*mean, SD*)	8.8 (0.8)	8.7 (0.6)	*p* = 0.937^a^

Categorical data was answered with “yes”

*TXA* tranexamic acid, *kg* kilo gram, *bpm* beats per minute, *SD* standard deviation, *mmHg* millimeter of mercury, *mmol/L* millimoles per liter

^a^Independent t test, ^b^Chi-squared test, ^c^Fisher exact test

### Primary endpoint

During surgery, 34/49 (69%) patients in the TXA-group needed hemostatic clips for active bleeding at the staple line versus 43/52 (83%) patients in the placebo-group (Relative Risk (RR): 0.84 [95% CI 0.67 to 1.05], *p* = 0.161). There was no significant difference in the mean number of clips used between both groups (3.5 versus 4.2; between-group difference 0.18 [95% CI -0.18 to 0.70], *p* = 0.377) (Table 2). There was no difference in the utilization of hemostatic clip use among surgeons, this is described in greater detail in supplementary file 3.

**Table 2:** Peroperative outcomes

	TXA (*n* = 49)	Placebo (*n* = 52)	Between-group difference (95% CI) or RR (95% CI)	*p*-value
Peroperative hemostatic clips used	34 (69.4%)	43 (82.7%)	RR: 0.84 (0.67 to 1.05)	*p* = 0.161^b^
Number of peroperative hemostatic clips used (*mean, SD*)	3.54 (2.7)	4.17 (2.4)	0.18 (− 0.18 to 0.70)	*p* = 0.377^a^
Fibrin sealant use peroperative	19 (38.8%)	23 (44.2%)	RR: 0.88 (0.55 to 1.40)	*p* = 0.687^b^
Staple device Cartridge GOLD (3.0 mm) Cartridge BLUE (2.4 mm) Cartridge GREEN (3.4 mm)	49 (100%) 49 (100%) 4 (8.9%)	52 (100%) 52 (100%) 5 (9.6%)		*p* = 1.000^c^
Blood loss (milliliter) (*mean, SD*)	0	0	UTD	UTD
Length of surgery (minutes) (*mean, SD*)	34.1 (11.8)	34.8 (8.7)	0.71 (− 4.80 to 3.39)	*p* = 0.733^a^

Categorical data was answered with “yes”

*TXA* tranexamic acid, *CI* confidence interval, *RR* relative risk, *SD* standard deviation, *UTD* unable to determine, *mm* millimeter. ^a^Independent t test; ^b^Chi-squared test; ^c^Fisher exact test

*Indicates statistical significance (*p* < .05)

### Secondary endpoints

**Fig. 2:**
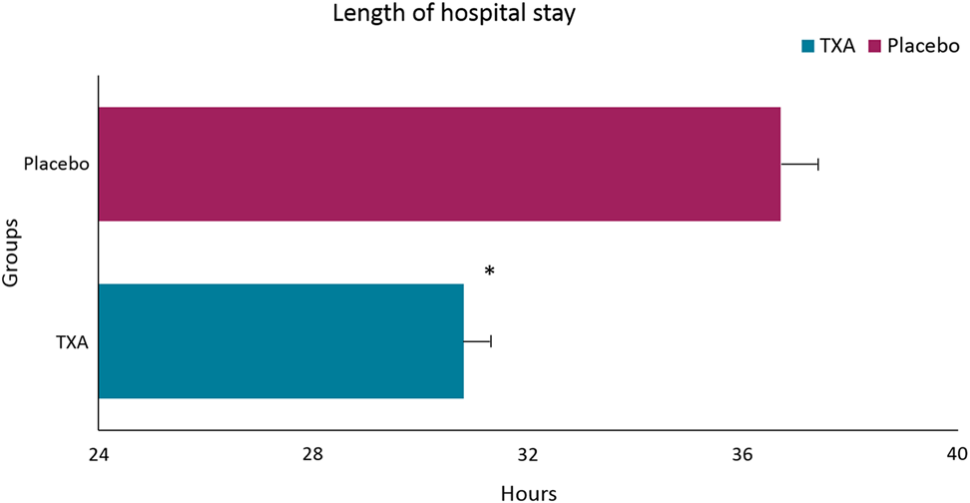
Bar chart of length of hospital stay between the TXA-group and placebo-group. All values are mean; whiskers show standard error of the mean. *TXA* tranexamic acid. *Indicates statistical significance (*p* < .05)

Table 2 presents the peroperative outcomes. No differences were seen in the rate of fibrin sealant use (19 (38.8%) versus 23 (44.2%); RR: 0.88 [95% CI 0.55 to 1.40], *p* = 0.687) and blood loss (0 versus 0, UTD). Table 3 displays the postoperative outcomes. The TXA-group demonstrated a significantly smaller decrease in hemoglobin versus the placebo-group (0.55 mmol/L versus 0.80 mmol/L; between-group difference, 0.25 mmol/L [95% CI 0.05 to 0.44], *p *= 0.013). Results were comparable after correction for LOP in a sensitivity analysis. Patients who had postoperative vomiting (*n* = 14) or a major complication (*n* = 1) received > 500-cc NaCl after their postoperative hemoglobin levels had been determined. Change in heart rate was significantly lower in the TXA-group versus the placebo-group (− 4.6 bpm versus 2.5 bpm; between-group difference, 7.1 bpm [95% CI 1.53 to 12.69], *p* = 0.013), with comparable results after correction for pain and LOP in a sensitivity analysis. Seven (14.3%) patients in the TXA-group had extra postoperative hemoglobin monitoring versus seventeen (32.7%) in the placebo-group (RR: 0.44 [95% CI 0.20 to 0.96], *p* = 0.037). In the TXA-group, there were no postoperative hemorrhage (major complications) requiring intervention, compared with one patient in the placebo-group who underwent radiological drainage of an infected hematoma originating from a staple line bleeding. One (2.0%) patient had a minor complication in the TXA-group (≥ 1.5 points hemoglobin decrease) versus nine (17.3%) in the placebo-group (hematemesis *n* = 1, melena *n* = 1, infected abdominal wall hematoma *n* = 1, and TXA for ≥ 1.5 points hemoglobin decrease *n* = 7; (RR: 0.12 [95% CI 0.02 to 0.90], *p* = 0.016)). There was no statistically significant association between the number of hemostatic clips and the odds of postoperative hemorrhage-related complications (odds ratio: 1.03, [95% CI 0.55 to 1.17], *p* = 0.674). Results for minor complications were comparable after correction for LOP, surgeon, and color cartridge in a sensitivity analysis (see supplementary file 3). Figure 2 shows a significantly shorter stay in the TXA-group versus the placebo-group (mean hours; 30.8 versus 36.7, between-group difference 5.9 [95% CI -10.5 to − 1.3] *p* = 0.013). Four patients (8.2%) in the TXA-group were discharged ≥ 36 h after surgery (nausea *n* = 2, vomiting *n* = 1, and pain *n* = 1). In the placebo-group eleven patients (21.2%) were discharged ≥ 36 h after surgery (nausea *n* = 4, vomiting *n* = 2, pain *n* = 1, hemoglobin decrease *n* = 2, (irregular) tachycardia for which an electrocardiogram was necessary *n* = 1, and infected staple line hematoma *n* = 1). Peroperative: use of linear stapler cartridge and LOP and postoperative: pain, general complications, and side effects of TXA were not significantly different between both groups. No VTE and mortality were reported. Primary and secondary outcomes are presented in more detail in Tables 2 and 3.

**Table 3:** Postoperative outcomes

	TXA (*n* = 49)	Placebo (*n* = 52)	Between-group difference (95% CI) or RR (95% CI)	*p*-value
Decrease in Hb level (mmol/L) (*mean, SD*)	− 0.55 (0.48)	− 0.80 (0.50)	0.25 (0.05 to 0.44)	*p* = 0.013^a^*
Increase in heart rate (bpm) (*mean, SD*)	− 4.6 (13.5)	2.5 (14.8)	7.11 (1.53 to 12.69)	*p* = 0.013^a^*
Pain (numeric rating scale) *(mean, SD)*	3.6 (1.9)	4.0 (1.7)	− 0.41 (− 1.11 to 0.30)	*p* = 0.256^a^
Intervention for hemorrhage	0 (0%)	1 (1.9%)	UTD	UTD
Patients with extra Hb monitoring	7 (14.3%)	17 (32.7%)	RR: 0.44 (0.20 to 0.96)	*p* = 0.037^b^*
Minor complications due to hemorrhage	1 (2.0%)	9 (17.3%)	RR: 0.12 (0.02 to 0.90)	*p* = 0.016^c^*
Hematemesis	0 (0%)	1 (1.9%)	UTD	UTD
Melena	0 (0%)	1 (1.9%)	UTD	UTD
TXA for decrease of > 1.5 Hb	1 (2.0%)	7 (13.5%)^d^	RR: 0.15 (0.02 to 1.19)	*p* = 0.060^c^
Infected abdominal wall hematoma	0 (0%)	1 (3.8%)	UTD	UTD
General complications	3 (6.1%)	10 (19.2%)	RR: 0.32 (0.09 to 1.09)	*p* = 0.073^c^
Length of stay (hours) *(mean, SD)*	30.8 (6.3)	34.7 (15.3)	− 5.9 (− 10.5 to − 1.3)	*p* = 0.013^a^*
Possible side effects TXA				
VTE	0 (0%)	0 (0%)	UTD	UTD
Nausea	21 (42.9)	21 (40.4)	RR: 1.06 (0.67 to 1.69)	*p* = 0.842^b^
Vomiting	8 (16.3%)	6 (11.5%)	RR: 1.70 (0.60 to 4.84)	*p* = 0.381^b^
Systolic bp (mmHg) *(mean, SD)*	135.7(16.5)	138.8 (17.7)	− 3.09 (− 9.88 to 3.70)	*p* = 0.368^a^
Diastolic bp (mmHg) *(mean, SD)*	72.5 (9.4)	73.3 (10.5)	− 0.80 (− 4.74 to 3.14)	*p* = 0.687^a^
Mortality	0 (0%)	0 (0%)	UTD	UTD

Categorical data were answered with “yes”

*TXA* tranexamic acid, *CI*, confidence interval, *RR* relative risk, *mmol/L* millimoles per liter, *SD* standard deviation, *bpm* beats per minute, *Hb* hemoglobin, *bp* blood pressure, *mmHg* millimeter of mercury, *VTE* venous thrombotic events, *UTD* unable to determine

^a^Independent t test; ^b^Chi-squared test; ^c^Fisher exact test. ^d^One patient is the patient with melena; this patient is counted once in the total number of complications

*Indicates statistical significance (*p* < .05)

## Discussion

In the present study, TXA did not show a significant reduction in the use of hemostatic clips. However, peroperative administration of TXA in patients undergoing SG was associated with significant positive effects on clinical parameters, and minor complications correlated with hemorrhage and LOS, without increasing side effects, such as the risk of VTE.

To the best of our knowledge, this is the first randomized controlled trial investigating the effectiveness of peroperative administration of TXA in metabolic surgery. In other surgical fields, clinical trials have shown positive effects of TXA on all-cause mortality, reduction of blood loss, and a reduction risk of reoperations, without an increase of VTE [15, 25–27]. The current incidence of VTE in patients undergoing metabolic surgery is relatively low, likely as a result of standard antithrombotic prophylaxis. Moreover, administration of TXA in metabolic and abdominal surgery does not seem to be associated with VTE [8, 25].

The available evidence of TXA in metabolic surgery comes from non-randomized and retrospective studies, showing less staple line bleedings in SG, low reoperation rates, and no increase in VTE [8, 18]. In the present study, hemostatic clips were used less frequently in patients receiving TXA. This difference was not statistically significant, whereas a non-randomized study in patients undergoing SG showed significantly less staple line bleeding points (19 versus 46, *p* < 0.05) requiring hemostatic stitches in patients receiving TXA versus patients without TXA [18]. The study might be underpowered to show a statistically significant difference in administration of TXA and the use of clips. Furthermore, no significant association was observed between the number of hemostatic clips used and postoperative complications. The literature lacks consensus in this regard, as some studies observed a reduction in postoperative bleeding after SLR, while others show no correlation and no cost-effective benefits [28–31]. Another important factor is staple height. Theories differ, some suggest that a tighter staple line reduces bleeding risk, while others caution that excessive tightness may have a paradoxical effect, resulting in tissue crush which increases bleeding risk. This study found no significant difference in staple height between both groups. The influence of staple height should be further investigated.

Retrospective studies have shown a decrease in hemoglobin and an increase in heart rate as early signs of hemorrhage after metabolic surgery [32–35]. In the present study, changes in both parameters were in favor of the TXA-group. A postoperative decrease in heart rate was seen in the TXA-group. One possible explanation is that patients who underwent successful surgery might experience less stress than preoperatively. Moreover, fewer minor complications due to hemorrhage occurred in the TXA-group. Although the differences in these prognostic factors did not translate into a significant reduction of hemorrhage and the difference in hemoglobin decrease was small, these findings might suggest a beneficial effect of TXA. However, further research is needed to gather more evidence and to evaluate the clinical relevance of this effect. In this study, patients who required more than 500 cc NaCl received it the day following their initial administration, after hemoglobin monitoring. However, if excess NaCl is administered beyond the protocolized volume before monitoring, such as in cases of immediate post-surgical major hemorrhage, it should be considered a potential covariate in the analysis.

As hypothesized, interventions for hemorrhage were rare, as the (limited) sample size was based on reduced use of clips for peroperative bleeding points. One radiological intervention was performed in the placebo-group, due to an infected hematoma originating from the staple line, compared to none in the TXA-group. This reported hemorrhage rates are in line with larger series (2–3%) [6, 7]. To establish a potential beneficial effect of TXA on hemorrhage requiring intervention, a large randomized controlled trial is needed. Such a trial preformed in hysterectomy patients showed a significantly reduced risk of reoperations in the TXA-group (2/165 (1.2%) versus 9/167 (5.4%), *p* = 0.034) [36].

TXA is an inexpensive drug and might be of economic value [18, 37]. Although a formal cost-effectiveness analysis has not been performed, LOS was significantly shorter in the TXA-group. This may not have impact on surgery with an overnight admission, but could be beneficial in day-care surgery, which is increasingly performed.

This study had several limitations, including the fact that it was conducted in a single institution. Nevertheless, the double-blinded randomized design, in which all operations were performed by expert bariatric surgeons, makes the results widely applicable. Secondly, the primary endpoint consisted of factors indirectly associated with hemorrhage. The definition of this endpoint was chosen in order to limit the sample size and minimize the number of patients exposed to potential risks, as this is the first randomized trial investigating TXA in metabolic surgery. Thirdly, the study might have been underpowered to show a reduction in use of clips. Based on the current results, a sample size of 316 would have been required to demonstrate a statistically significant reduction in hemostatic clip use between TXA and placebo (*α* = 5%, power = 80%, TXA = 30.6%, placebo = 17.3%). Fourthly, thrombosis prophylaxis was only administered postoperatively, as this is standard of care in the Netherlands [22]. However, the absence of preoperative thrombosis prophylaxis did not result in an increase in thrombotic events and is supported by existing data [10, 22]. Finally, surgeons’ emphasis on staple line control may have biased the primary endpoint, resulting in increased use of hemostatic clip. Although, the intra-operative hemostasis was protocolized and consistently implemented by each surgeon (see supplementary file 2 and 3). We have designed a new trial (registration number: R22.102, ClinicalTrials.gov NCT05464394) to further investigate the potential of TXA in reducing postoperative hemorrhage, with postoperative interventions as primary outcome. This trial has been approved by the Medical Research Ethics Committees on February 7th, 2023. Additionally, given the increased bleeding risk associated with anticoagulant use [7], a study to assess the efficacy and safety of TXA in patients on anticoagulants would be valuable provided that safety measures are in place.

## Conclusion

This study could not demonstrate a statistically significant difference in use of hemostatic clips and in major complications after peroperative administration of TXA. However, TXA seems to have a positive effect on postoperative hemoglobin, heart rate, minor complications due to hemorrhage and LOS in patients undergoing SG, without increasing VTE rate, and other side effects. A limitation of this study is the primary endpoint, reduction in hemostatic clips. To investigate whether TXA can reduce postoperative hemorrhages, we are conducting a larger randomized controlled trial with postoperative interventions as primary outcome.

### Supplementary Information

Below is the link to the electronic supplementary material.

Supplementary file1 (PDF 393 KB)

Supplementary file2 (DOCX 13 KB)

Supplementary file3 (DOCX 14 KB)
