# Supplementary material for: Peroperative administration of tranexamic acid in sleeve gastrectomy to reduce hemorrhage: a double-blind randomized controlled trial
Source: Surg Endosc. 2023 Jul 3;37(10):7455–63. doi: 10.1007/s00464-023-10232-5 (PMC10520143; doi:10.1007/s00464-023-10232-5)
Supplement: Supplementary file 1 — Supplementary file1 (PDF 393 KB) [file 464_2023_10232_MOESM1_ESM.pdf]

STUDY PROTOCOL: PEROPERATIVE TRANEXAMIC ACID IN BARIATRIC FAST TRACK SURGERY TO REDUCE HEMORRAGE RATES

(July 2019)

TABLE OF CONTENTS

1. INTRODUCTION AND RATIONALE 6
2. OBJECTIVES 6
3. STUDY DESIGN 7
4. STUDY POPULATION 7
4.1 Population (base) 7
4.2 Inclusion criteria 7
4.3 Exclusion criteria 7
4.4 Sample size calculation 7
5. TREATMENT OF SUBJECTS 8
5.1 Investigational product/treatment 8
5.2 Use of co-intervention (if applicable) 8
5.3 Escape medication (if applicable) 8
6. INVESTIGATIONAL PRODUCT 8
6.1 Name and description of investigational product(s) 8
6.2 Summary of findings from non-clinical studies 8
6.3 Summary of findings from clinical studies 8
6.4 Summary of known and potential risks and benefits 8
6.5 Description and justification of route of administration and dosage 9
6.6 Dosages, dosage modifications and method of administration 9
6.7 Preparation and labelling of Investigational Medicinal Product 9
6.8 Drug accountability 9
7. NON-INVESTIGATIONAL PRODUCT 9
7.1 Name and description of non-investigational product(s) 9
7.2 Summary of findings from non-clinical studies 9
7.3 Summary of findings from clinical studies 10
7.4 Summary of known and potential risks and benefits 10
7.5 Description and justification of route of administration and dosage 10
7.6 Dosages, dosage modifications and method of administration 10
7.7 Preparation and labelling of Non Investigational Medicinal Product 10
7.8 Drug accountability 10
8. METHODS 10
8.1 Study parameters/endpoints 10
8.1.1 Main study parameter/endpoint 10
8.1.2 Secondary study parameters/endpoints (if applicable) 10
8.1.3 Other study parameters (if applicable) 10
8.2 Randomisation, blinding and treatment allocation 10
8.3 Study procedures 11
8.4 Withdrawal of individual subjects 11
8.4.1 Specific criteria for withdrawal (if applicable) 11
8.5 Replacement of individual subjects after withdrawal 11
8.6 Follow-up of subjects withdrawn from treatment 11

8.7 Premature termination of the study....11
9. SAFETY REPORTING....11
9.1 Temporary halt for reasons of subject safety....11
9.2 AEs, SAEs and SUSARs....12
9.2.1 Adverse events (AEs)....12
9.2.2 Serious adverse events (SAEs)....12
9.2.3 Suspected unexpected serious adverse reactions (SUSARs)....13
9.3 Annual safety report....14
9.4 Follow-up of adverse events....14
9.5 Data Safety Monitoring Board (DSMB) / Safety Committee Not applicable.....14
10. STATISTICAL ANALYSIS....14
10.1 Primary study parameter(s)....14
10.2 Secondary study parameter(s)....15
10.3 Other study parameters....15
10.4 Interim analysis (if applicable)....15
11. ETHICAL CONSIDERATIONS....15
11.1 Regulation statement....15
11.2 Recruitment and consent....15
11.3 Objection by minors or incapacitated subjects (if applicable)....15
11.4 Benefits and risks assessment, group relatedness....15
11.5 Compensation for injury....16
11.6 Incentives (if applicable)....16
12. ADMINISTRATIVE ASPECTS, MONITORING AND PUBLICATION....16
12.1 Handling and storage of data and documents....16
12.2 Monitoring and Quality Assurance....16
12.3 Amendments....17
12.4 Annual progress report....17
12.5 Temporary halt and (prematurely) end of study report....17
12.6 Public disclosure and publication policy....18
13. STRUCTURED RISK ANALYSIS....18
13.1 Potential issues of concern....18
13.2 Synthesis....18
14. REFERENCES....18

LIST OF ABBREVIATIONS AND RELEVANT DEFINITIONS

**ABR**	**General Assessment and Registration form (ABR form), the application form that is required for submission to the accredited Ethics Committee; in Dutch: Algemeen Beoordelings- en Registratieformulier (ABR-formulier)**
**AE**	**Adverse Event**
**AR**	**Adverse Reaction**
**CA**	**Competent Authority**
**CCMO**	**Central Committee on Research Involving Human Subjects; in Dutch: Centrale Commissie Mensgebonden Onderzoek**
**CV**	**Curriculum Vitae**
**DSMB**	**Data Safety Monitoring Board**
**ERABS**	**Enhanced Recovery After Bariatric Surgery**
**EU**	**European Union**
**EudraCT**	**European drug regulatory affairs Clinical Trials**
**GCP**	**Good Clinical Practice**
**IB**	**Investigator's Brochure**
**IC**	**Informed Consent**
**IMP**	**Investigational Medicinal Product**
**IMPD**	**Investigational Medicinal Product Dossier**
**METC**	**Medical research ethics committee (MREC); in Dutch: medisch-ethische toetsingscommissie (METC)**
**LRYGB**	**Laparoscopic Roux-en-Y Gastric Bypass**
**LSG**	**Laparoscopic Sleeve Gastrectomy**
**(S)AE**	**(Serious) Adverse Event**
**Sponsor**	**The sponsor is the party that commissions the organisation or performance of the research, for example a pharmaceutical company, academic hospital, scientific organisation or investigator. A party that provides funding for a study but does not commission it is not regarded as the sponsor, but referred to as a subsidising party.**
**SUSAR**	**Suspected Unexpected Serious Adverse Reaction**
**TA**	**Tranexamic acid**
**WMO**	**Medical Research Involving Human Subjects Act; in Dutch: Wet Medisch-wetenschappelijk Onderzoek met Mensen**

SUMMARY

Rationale: Fast-track protocols are frequently used in bariatric surgery and often include short-term thromboprophylaxis and short length of hospital stay. These treatment strategies may negatively affect the occurrence and diagnosis of postoperative hemorrhage. Over the years, the rates of venous thromboembolic events (VTE) decrease, while there seems to be an increase in the occurrence of postoperative hemorrhage. Tranexamic acid (TA) is a plasminogen inhibitor which inhibits fibrinolysis. Peroperative administration of TA may lower the incidence of postoperative hemorrhage.
Objective: This trial aims to investigate if peroperative administration of TA can reduce the peroperative and postoperative hemorrhage rates in laparoscopic sleeve gastrectomy.
Study design: This is a double-blind, single center randomized controlled trial.
Study population: Patients eligible for bariatric surgery according to the IFSO guidelines and undergoing a laparoscopic sleeve gastrectomy. Patients unwilling to give informed consent, patients with a medical history of bleeding or VTE and patients that use therapeutic anticoagulants will be excluded.
Intervention (if applicable): Patients are randomized between 2 groups: 1) Single dose of 1500 mg TA to be administered during induction of the procedure by anesthesiologist, and 2) Standard protocol with administration of placebo infusion.
Main study parameters/endpoints: Primary outcome measure is peroperative use of hemostatic clips. Secondary outcome measures are the decrease in hemoglobin after the procedure, rates of postoperative hemorrhage (i.e. hemorrhage needing administration of packed cells or a surgical or radiological re-intervention) and rates of VTE.
Nature and extent of the burden and risks associated with participation, benefit and group relatedness: All patients will be required to undergo one additional blood drawing in the week prior to the procedure which will be performed at their weighing appointment, meaning that this will not require an extra hospital visit. TA has very little side effects. Therefore we conclude that the expected benefits of the intervention outweigh the minor risks involved.

1. INTRODUCTION AND RATIONALE

Because of the increasing rates of morbid obesity and herewith increasing requests for bariatric surgery, the popularity of fast-track protocols is increasing. These Enhanced Recovery After Bariatric Surgery (ERABS) protocols often include short-term thromboprophylaxis and short length of hospital stay (LOS), with the aim to discharge patients on the first postoperative day(1, 2). Even though ERABS has many positive outcomes, it may also negatively affect the diagnosis of postoperative hemorrhage due to the short time window for diagnosing postoperative complications.

Most current literature states that postoperative hemorrhage occurs in 2.0% of patients undergoing laparoscopic sleeve gastrectomy (LSG) and in 1.5-3.1% of patients undergoing a laparoscopic Roux-en-Y gastric bypass (LRYGB)(3, 4). It is described that over the years, there seems to be an increase in the occurrence of postoperative hemorrhage, while the risk of venous thromboembolic events (VTE) is decreasing(5). In our center, overall postoperative hemorrhage rates of LRYGB and LSG have been stable since 2014, with more hemorrhage occurring after a LSG (2.2%) than after a LRYGB (1.3%). The rates of peroperative hemorrhage requiring hemostatic clips was 63.3% between January 2017 and March 2019. The rate of diagnosed VTE within 3 months postoperative is 0.05% since 2014 in this center.

Tranexamic acid (TA) is a plasminogen inhibitor that can be used to inhibit fibrinolysis during or after surgery and thus minimize the risk of developing perioperative hemorrhage. Little is known about the use of TA in bariatric surgery. Nevertheless, in several other surgical areas such as for coronary-artery surgery and arthroplasty, perioperative administration of TA has proven its value in preventing postoperative hemorrhage (6-8). Until now, the use of TA to prevent postoperative hemorrhage in bariatric surgery is once described by Hussain et al.(9). The authors performed a meta-analysis of administration of TA during laparoscopic sleeve gastrectomies and concluded that administration of TA is a simple yet effective way to lower the rates of staple line hemorrhage, while shortening the length of surgery. In the end of 2018, Klaassen et al described that reoperations may be prevented due to postoperative administration of TA in case of a suspicion of an already present hemorrhage(10).

Because of the serious risks of postoperative hemorrhage, the need for preventive measures in bariatric fast-track surgery is increasing. This trial aims to investigate if peroperative administration of TA can reduce the postoperative hemorrhage rates in laparoscopic sleeve gastrectomy.

2. OBJECTIVES

Primary Objective: Does the peroperative administration of TA lead to lesser use of peroperative extra hemostatic clips?

Secondary Objective(s): Does the peroperative administration of TA lead to a smaller hemoglobin decrease 1 day postoperatively and a lower reoperation rate within 30 days postoperative due to hemorrhage, or an increase in venous thromboembolic events within 3 months postoperative?

3. STUDY DESIGN

This is a double-blind, single center randomized placebo-controlled trial that will last for approximately 1 years, depending on the speed of inclusion. We aim to include 100 patients.

Patients are equally randomized between 2 groups: 1) Single dose of TA to be administered during induction of the procedure by anesthesiologist, and 2) Standard protocol with administration of a placebo (sodium chloride).

4. STUDY POPULATION

4.1 Population (base)

All patients found suitable for bariatric surgery according to the international guidelines and undergoing a laparoscopic sleeve gastrectomy (LSG) will be asked to participate in the study. All patients will follow the hospital's Enhanced Recovery After Bariatric Surgery (ERABS) protocol, which includes chemical thromboprophylaxis (Dalteparine 1 dd 5000 IE during hospital admission).

4.2 Inclusion criteria

In order to be eligible to participate in this study, a subject must meet all of the following criteria: Primary bariatric procedure; good command of the Dutch or English language.

4.3 Exclusion criteria

A potential subject who meets any of the following criteria will be excluded from participation in this study: Patients unwilling to give informed consent, patients with a medical history of bleeding or VTE and patients who use therapeutic anticoagulants. Patients will also be excluded in case of peroperative arterial bleeding or (iatrogenic) bleeding coming from surrounding organs or vascular structures such as the liver or the spleen.

4.4 Sample size calculation

The performed power analysis (power= 80%, alfa=5% two-sided) calculated a required sample size of 2x36=72 patients. The analysis was based on an expected 50% decrease of the percentage of patients for whom placement of hemostatic staples was required peroperatively.

5. TREATMENT OF SUBJECTS

5.1 Investigational product/treatment

In the intervention group, patients will be administered tranexamic acid 1500 mg intravenous at induction of the procedure. In the control group, patients will receive a placebo infusion with sodium chloride.

5.2 Use of co-intervention (if applicable)

Not applicable.

5.3 Escape medication (if applicable)

Not applicable.

6. INVESTIGATIONAL PRODUCT

6.1 Name and description of investigational product(s)

Tranexamic acid injections, 100 mg/ml, ampoule 5 ml. It is a competitive inhibitor of plasminogen and therefore of the conversion into plasmin in the fibrinolytic system. In high doses, tranexamic acid is a non-competitive plasmin inhibitor(11).

6.2 Summary of findings from non-clinical studies

The clinical studies as mentioned in paragraph 6.3 provide the most up-to-date and applicable information.

For details see the IMPD of tranexamic acid.

6.3 Summary of findings from clinical studies

Tranexamic acid (TA) is a plasminogen inhibitor that can be used to inhibit fibrinolysis during or after surgery and thus minimize the risk of developing perioperative hemorrhage. Little is known about the use of TA in bariatric surgery. Nevertheless, in several other surgical areas such as for coronary-artery surgery and arthroplasty, perioperative administration of TA has proven its value in preventing postoperative hemorrhage (6-8). Up until now, the use of TA to prevent postoperative hemorrhage in bariatric surgery is once described by Hussain et al.(9). The authors performed a meta-analysis of administration of TA during laparoscopic sleeve gastrectomies and concluded that administration of TA is a simple yet effective way to lower the rates of staple line hemorrhage, while shortening the length of surgery. In the end of 2018, Klaassen et al described that reoperations may be prevented due to postoperative administration of TA in case of a suspicion of an already present hemorrhage(10).

6.4 Summary of known and potential risks and benefits

Side effects of tranexamic acid include dose-dependent gastrointestinal complaints in 1-10% (nausea, vomiting, diarrhea); allergic skin reactions in 0.1-1%; and

thrombocytopenia, thromboembolic events, blindness, color blindness and other visual disorders in 0.01-0.1%.
Benefits of tranexamic acid include the prevention of perioperative hemorrhage in several surgical areas (6-9) and treatment of postoperative hemorrhage and herewith prevention of reoperation after bariatric surgery(10).

6.5 Description and justification of route of administration and dosage

For general (non-topical) fibrinolysis in adults, tranexamic acid is administered intravenously in a dosage of 15 mg/kg bodyweight in a solution with sodium chloride. Administration can be repeated every 6-8 hours. In our study population, body weight varies between 100 and 200 kg. Tranexamic acid has a small volume of distribution (0.12-0.17 mg/kg). The acid part of tranexamic acid is in a fysiological pH depronotated and the amino part is protonated. Therefore, we expect that the molecule will mostly be present in watery environment and will not spread to the fatty tissue. Therefore, we expect that a dosage above 1500 mg will not be of additional value.

6.6 Dosages, dosage modifications and method of administration

A set dose of 1500 mg will be used, based on the study population with morbid obesity. It will be administered intravenous dissolved in 100 ml sodium chloride 0.9% in a time frame of 15-30 minutes, with a maximum of 100 mg/min.

6.7 Preparation and labelling of Investigational Medicinal Product

The Investigational Medicinal Product will be ordered according to local procedures at the Department of Pharmacy in the [redacted] and will be brought to the OR by the principal investigator. Preparation and labelling of the investigational medicinal products is done according to the relevant GMP guidelines for Good Manufacturing Practice by the hospital's pharmacy.

6.8 Drug accountability

The local Department of Pharmacy can provide all the medication that is required for this trial.

7. NON-INVESTIGATIONAL PRODUCT

7.1 Name and description of non-investigational product(s)

Placebo, saline injection fluid produced by Baxter.

7.2 Summary of findings from non-clinical studies

The placebo contains a saline solution which has no effect on fibrinolysis.

7.3 Summary of findings from clinical studies

7.4 Summary of known and potential risks and benefits

7.5 Description and justification of route of administration and dosage

7.6 Dosages, dosage modifications and method of administration

100 ml sodium chloride 0.9% will be administered in a time frame of 15-30 minutes.

7.7 Preparation and labelling of Non Investigational Medicinal Product

The non-investigational product will be ordered according to local procedures at the Department of Pharmacy in the [redacted] and will be brought to the OR by the principal investigator. Preparation and labelling of the non-investigational products is done according to the relevant GMP guidelines for Good Manufacturing Practice by the hospital’s pharmacy.

7.8 Drug accountability

The local Department of Pharmacy can provide all the medication that is required for this trial.

8. METHODS

8.1 Study parameters/endpoints

8.1.1 Main study parameter/endpoint

Primary outcome measure is peroperative use of hemostatic staples.

8.1.2 Secondary study parameters/endpoints (if applicable)

Secondary outcome measures are the decrease in hemoglobin after the procedure at 1 day postoperative, rates of postoperative hemorrhage (i.e. hemorrhage needing administration of packed cells or a surgical or radiological re-intervention) within 30 days postoperative and rates of VTE within 3 months postoperative.

8.1.3 Other study parameters (if applicable)

Comorbidities such as hypertension, diabetes en dyslipidemia will be taken into account. Also, the peroperative blood pressure and the duration of surgery will be registered.

8.2 Randomisation, blinding and treatment allocation

After obtaining informed consent at the outpatient clinic of the surgical or anesthesiological department, patients will be randomized into 1 of the 2 groups using Variable Block Randomization software by Ciwit B.V. (Castor EDC). Patients will either receive tranexamic acid or placebo (sodium chloride) during induction. Patient, surgical team and anesthesiological team will be blinded for the treatment, as the hospital pharmacy will prepare the infusion bags for each individual patient.

Availability of the patient's individualized infusion bag will be checked at the Time Out Procedure (TOP). The coordinating researcher is unblinded in order to be able to consult the treatment arm in case of emergency.

8.3 Study procedures

An extra blood sample (1 EDTA tube) is obtained in the week preoperative by venipuncture. From this blood sample, hemoglobin tests are performed in the hospital's laboratory. Postoperative hemoglobin value will be obtained from the blood sample that is performed in all bariatric patients on the first postoperative day. Information on peroperative use of hemostatic staples, administration of packed cells, postoperative tranexamic acid, rates of postoperative hemorrhage and VTE are obtained from the electronical patient file. Routine venous duplex ultrasound will not be performed. Therefore, only clinically significant VTEs are registered.

8.4 Withdrawal of individual subjects

Subjects can leave the study at any time for any reason if they wish to do so without any consequences. The investigator can decide to withdraw a subject from the study for urgent medical reasons, for example such as mentioned in the exclusion criteria.

8.4.1 Specific criteria for withdrawal (if applicable)

Not applicable.

8.5 Replacement of individual subjects after withdrawal

After withdrawal, individual subjects will be replaced by including new patients at the outpatient clinic to guarantee the needed number for sufficient power for analysis.

8.6 Follow-up of subjects withdrawn from treatment

All subjects withdrawn from treatment, will be followed-up according to the bariatric surgery guidelines.

8.7 Premature termination of the study

In case of substantially more (serious) adverse events in one of each groups that are correlated to tranexamic acid, the study can be terminated prematurely in consultation with the METC.

9. SAFETY REPORTING

9.1 Temporary halt for reasons of subject safety

In accordance to section 10, subsection 4, of the WMO, the sponsor will suspend the study if there is sufficient ground that continuation of the study will jeopardise subject health or safety. The sponsor will notify the accredited METC without undue delay of a

temporary halt including the reason for such an action. The study will be suspended pending a further positive decision by the accredited METC. The investigator will take care that all subjects are kept informed.

9.2 AEs, SAEs and SUSARs

9.2.1 Adverse events (AEs)

Adverse events are defined as any undesirable experience occurring to a subject during the study, whether or not considered related to the experimental intervention. All adverse events reported spontaneously by the subject or observed by the investigator or his staff will be recorded.

9.2.2 Serious adverse events (SAEs)

A serious adverse event is any untoward medical occurrence or effect that

- results in death;
- is life threatening (at the time of the event);
- requires hospitalisation or prolongation of existing inpatients’ hospitalisation;
- results in persistent or significant disability or incapacity;
- is a congenital anomaly or birth defect; or
- any other important medical event that did not result in any of the outcomes listed above due to medical or surgical intervention but could have been based upon appropriate judgement by the investigator.

An elective hospital admission will not be considered as a serious adverse event.

The investigator will report all SAEs that occur within 3 months postoperative to the sponsor without undue delay after obtaining knowledge of the events.

The sponsor will report the SAEs through the web portal *ToetsingOnline* to the accredited METC that approved the protocol, within 7 days of first knowledge for SAEs that result in death or are life threatening followed by a period of maximum of 8 days to complete the initial preliminary report. All other SAEs will be reported within a period of maximum 15 days after the sponsor has first knowledge of the serious adverse events.

9.2.3 Suspected unexpected serious adverse reactions (SUSARs)

Adverse reactions are all untoward and unintended responses to an investigational product related to any dose administered.

Unexpected adverse reactions are SUSARs if the following three conditions are met:

1. the event must be serious (see chapter 9.2.2);
2. there must be a certain degree of probability that the event is a harmful and an undesirable reaction to the medicinal product under investigation, regardless of the administered dose;
3. the adverse reaction must be unexpected, that is to say, the nature and severity of the adverse reaction are not in agreement with the product information as recorded in:
   - Summary of Product Characteristics (SPC) for an authorised medicinal product;
   - Investigator's Brochure for an unauthorised medicinal product.

The sponsor will report expedited the following SUSARs through the web portal *ToetsingOnline* to the METC:

- SUSARs that have arisen in the clinical trial that was assessed by the METC;
- SUSARs that have arisen in other clinical trials of the same sponsor and with the same medicinal product, and that could have consequences for the safety of the subjects involved in the clinical trial that was assessed by the METC.

The remaining SUSARs are recorded in an overview list (line-listing) that will be submitted once every half year to the METC. This line-listing provides an overview of all SUSARs from the study medicine, accompanied by a brief report highlighting the main points of concern.

The expedited reporting of SUSARs through the web portal Eudravigilance or ToetsingOnline is sufficient as notification to the competent authority.

The sponsor will report expedited all SUSARs to the competent authorities in other Member States, according to the requirements of the Member States.

The expedited reporting will occur not later than 15 days after the sponsor has first knowledge of the adverse reactions. For fatal or life threatening cases the term will be maximal 7 days for a preliminary report with another 8 days for completion of the report.

9.3 Annual safety report

In addition to the expedited reporting of SUSARs, the sponsor will submit, once a year throughout the clinical trial, a safety report to the accredited METC, competent authority, and competent authorities of the concerned Member States.

This safety report consists of:

- a list of all suspected (unexpected or expected) serious adverse reactions, along with an aggregated summary table of all reported serious adverse reactions, ordered by organ system, per study;
- a report concerning the safety of the subjects, consisting of a complete safety analysis and an evaluation of the balance between the efficacy and the harmfulness of the medicine under investigation.

9.4 Follow-up of adverse events

All AEs will be followed until they have abated, or until a stable situation has been reached. Depending on the event, follow up may require additional tests or medical procedures as indicated, and/or referral to the general physician or a medical specialist. SAEs need to be reported till end of study within the Netherlands, as defined in the protocol

9.5 Data Safety Monitoring Board (DSMB) / Safety Committee

Not applicable.

10. STATISTICAL ANALYSIS

Statistical analyses will be performed using IBM-SPSS version 24 (IBM Corporation, Armonk, New York, USA). Efforts will be made to prevent missing data by checking completeness of collected data. Missing data may be expected because of failed hemoglobin tests, or due to (less likely) unregistered administration of tranexamic acid or packed cells.

10.1 Primary study parameter(s)

Differences in use of hemostatic staples (yes/no) between the 2 groups will be determined using Chi-Square tests. The difference in hemoglobin decrease will be tested using multivariate linear regression analysis, correcting for comorbidities (hypertension, diabetes, dyslipidemia) and use of hemostatic staples. These same analyses will be performed to assess the differences in amount of patients needing to undergo a re-

intervention, but adding administration of postoperative tranexamic acid as a covariate. Results will be evaluated at a significance threshold of $p<0.05$ (two-sided).

10.2 Secondary study parameter(s)

Differences in rates of DVT (yes/no), PE (yes/no) and in rates of administration of postoperative tranexamic acid (yes/no) between the 2 groups will be calculated using Chi-Square (Fisher's Exact) tests.

10.3 Other study parameters

Not applicable.

10.4 Interim analysis (if applicable)

Not applicable.

11. ETHICAL CONSIDERATIONS

11.1 Regulation statement

The study will be conducted according to the principles of the Declaration of Helsinki (version 10, October 2013) and in accordance with the Medical Research Involving Human Subjects Act (WMO).

11.2 Recruitment and consent

All patients found suitable for bariatric surgery according to the international guidelines and undergoing a laparoscopic sleeve gastrectomy (LSG) will have received written information attached with the invitation letter for the appointment at the outpatient clinic. Once more, they will be informed by the doctor at the outpatient clinic of the department of surgery or anaesthesiology and if willing to participate, they will be asked to fill in the informed consent form. Patients will have one week to consider their decision.

11.3 Objection by minors or incapacitated subjects (if applicable)

Not applicable.

11.4 Benefits and risks assessment, group relatedness

The risk of the investigational treatment is possible experiencing side effects of tranexamic acid as mentioned in paragraph 6.4. Nevertheless, the bariatric procedure

itself will be the primary cause of gastrointestinal complains such as nausea and vomiting. The benefit of the treatment is the expected decrease in risk of postoperative hemorrhage, which is a very serious complication and can lead to extended length of hospital stay, re-interventions and (in very few cases) death.

11.5 Compensation for injury

The sponsor/investigator has a liability insurance which is in accordance with article 7 of the WMO.

The sponsor (also) has an insurance which is in accordance with the legal requirements in the Netherlands (Article 7 WMO). This insurance provides cover for damage to research subjects through injury or death caused by the study.
The insurance applies to the damage that becomes apparent during the study or within 4 years after the end of the study.

11.6 Incentives (if applicable)

Not applicable.

12. ADMINISTRATIVE ASPECTS, MONITORING AND PUBLICATION

12.1 Handling and storage of data and documents

Identification of participants will be protected by using study numbers non-traceable to patient’s identity. Only members of the research-team will have access to the databases with study data. Data will be kept for 20 years.

12.2 Monitoring and Quality Assurance

An independent monitor (quality officer), appointed by the sponsor, will monitor the study data according to Good Clinical Practice (GCP). The frequency of the monitoring is related to the risk of the investigation and here considered to be in the *minimal risk* category. For at least a selection of the subjects, Informed Consents will be checked. Source Data Verification will not be performed (checking if data from the Case Report Forms (research forms / questionnaires) matches with the source data (patient status, results, etc.)). The quality of data will be guaranteed by using the data management software Castor EDC. The monitor will check whether all (S)AEs and SUSARs are adequately reported within the timelines as required by law, the presence and correctness of the Informed consent forms, the delegation log and the data storage.

12.3 Amendments

A ‘substantial amendment’ is defined as an amendment to the terms of the METC application, or to the protocol or any other supporting documentation, that is likely to affect to a significant degree:

- the safety or physical or mental integrity of the subjects of the trial;
- the scientific value of the trial;
- the conduct or management of the trial; or
- the quality or safety of any intervention used in the trial.

All substantial amendments will be notified to the accredited METC and to the competent authority.

Non-substantial amendments will not be notified to the accredited METC and the competent authority, but will be recorded and filed by the sponsor.

12.4 Annual progress report

The investigator will submit a summary of the progress of the trial to the accredited METC once a year. Information will be provided on the date of inclusion of the first subject, numbers of subjects included and numbers of subjects that have completed the trial, serious adverse events/serious adverse reactions, other problems, and amendments.

12.5 Temporary halt and (prematurely) end of study report

The sponsor will notify the accredited METC and the competent authority of the end of the study within a period of 90 days. The end of the study is defined as 3 months after the surgery of the last patient.

The sponsor will notify the accredited METC immediately of a temporary halt of the study, including the reason of such an action.

In case the study is ended prematurely, the sponsor will notify the accredited METC and the competent authority within 15 days, including the reasons for the premature termination.

Within one year after the end of the study, the investigator will submit a final study report with the results of the study, including any publications/abstracts of the study, to the accredited METC and the Competent Authority.

12.6 Public disclosure and publication policy

The results of this pilot study will be published in peer reviewed journals. It will be the responsibility of the PI of the study to write down the results within a year of its completion. No material may be submitted for presentation and publication without prior review and a written approval by the study PI.

13. STRUCTURED RISK ANALYSIS

13.1 Potential issues of concern

Not applicable.

13.2 Synthesis

Since the use of tranexamic acid as prevention or treatment of postoperative hemorrhage has already been proven to be safe, chapter 13.2 is skipped.

Risks of the treatment are a slower postoperative recovery for patients in the intervention group, due to experiencing side effects of tranexamic acid. These side effects are described in paragraph 6.4 and occur in only a small percentage of patients. Furthermore, the most frequently observed side effects of TA are gastro-intestinal complaints, such as nausea and vomiting. These are complaints that patients usually experience after bariatric surgery and therefore the side effects that may be caused by the medication, seem to be of minor impact. Also, the advantage of expected less postoperative hemorrhage in our opinion outweighs the minor risks.

14. REFERENCES

1. Malczak P, Pisarska M, Piotr M, Wysocki M, Budzynski A, Pedziwiatr M. Enhanced Recovery after Bariatric Surgery: Systematic Review and Meta-Analysis. Obesity surgery. 2017;27(1):226-35.
2. Mannaerts GH, van Mil SR, Stepaniak PS, Dunkelgrun M, de Quelerij M, Verbrugge SJ, et al. Results of Implementing an Enhanced Recovery After Bariatric Surgery (ERABS) Protocol. Obesity surgery. 2016;26(2):303-12.
3. Zafar SN, Miller K, Felton J, Wise ES, Kligman M. Postoperative bleeding after laparoscopic Roux en Y gastric bypass: predictors and consequences. Surgical endoscopy. 2018.
4. Zellmer JD, Mathiason MA, Kallies KJ, Kothari SN. Is laparoscopic sleeve gastrectomy a lower risk bariatric procedure compared with laparoscopic Roux-en-Y gastric bypass? A meta-analysis. American journal of surgery. 2014;208(6):903-10; discussion 9-10.
5. Becattini C, Agnelli G, Manina G, Noya G, Rondelli F. Venous thromboembolism after laparoscopic bariatric surgery for morbid obesity: clinical burden and prevention. Surgery for obesity and related diseases : official journal of the American Society for Bariatric Surgery. 2012;8(1):108-15.

6. Kirsch JM, Bedi A, Horner N, Wiater JM, Pauzenberger L, Koueiter DM, et al. Tranexamic Acid in Shoulder Arthroplasty: A Systematic Review and Meta-Analysis. JBJS reviews. 2017;5(9):e3.
7. Meftah M, White PB, Siddiqi A, Siddappa VH, Kirschenbaum I. Tranexamic Acid Reduces Transfusion Rates in Obese Patients Undergoing Total Joint Arthroplasty. Surgical technology international. 2019;34.
8. Myles PS, Smith JA, Forbes A, Silbert B, Jayarajah M, Painter T, et al. Tranexamic Acid in Patients Undergoing Coronary-Artery Surgery. The New England journal of medicine. 2017;376(2):136-48.
9. Hussain A, Al-Shoek I, El-Hasani S. The Use of Tranexamic Acid in Sleeve Gastrectomy. Obesity surgery. 2017;27(1):198-9.
10. Klaassen RA, Selles CA, van den Berg JW, Poelman MM, van der Harst E. Tranexamic acid therapy for postoperative bleeding after bariatric surgery. BMC obesity. 2018;5:36.
11. Zorginstituut Nederland. Tranexaminezuur (bij fibrinolyse). Farmacotherapeutisch Kompas,. 2018.
