# Supplementary material for: Peroperative administration of tranexamic acid in sleeve gastrectomy to reduce hemorrhage: a double-blind randomized controlled trial
Source: Surg Endosc. 2023 Jul 3;37(10):7455–63. doi: 10.1007/s00464-023-10232-5 (PMC10520143; doi:10.1007/s00464-023-10232-5)
Supplement: Supplementary file 2 — Supplementary file2 (DOCX 13 KB) [file 464_2023_10232_MOESM2_ESM.docx]

Supplementary file 2

| Steps before placing hemostatic clips: | |
| --- | --- |
| ► | Staple gastric sleeve |
| ► | Remove nasogastric tube |
| ► | Remove sleeve from the abdomen |
| ► | Aim for normotension |
| ► | Decrease intraabdominal pressure to 12 mm Hg |
| ► | Inspection of staple line |
| ► | Place clips in case of ≥1 actively spraying bleeding spot |
| ► | Place fibrin sealant in case of ≥1 oozing bleeding spot |

Steps to be followed by the surgeon before placement of hemostatic clips. mmHg: millimeter of mercury
