# Supplementary material for: Peroperative administration of tranexamic acid in sleeve gastrectomy to reduce hemorrhage: a double-blind randomized controlled trial
Source: Surg Endosc. 2023 Jul 3;37(10):7455–63. doi: 10.1007/s00464-023-10232-5 (PMC10520143; doi:10.1007/s00464-023-10232-5)
Supplement: Supplementary file 3 — Supplementary file3 (DOCX 14 KB) [file 464_2023_10232_MOESM3_ESM.docx]

Supplementary file 3

Sensitivity analysis for hemoglobin, heart rate, minor complications, and hemoclip use per surgeon. ^a^Linear, ^b^logistic regression analysis, or Chi-Squared test were used when appropriate

Decrease in hemoglobin level was significantly less in the TXA-group versus the placebo-group (0.55mmol/L versus 0.80mmol/L; between-group difference, 0.25mmol/L [95% CI 0.05 to 0.44], p = 0.013). After correction^a^ for LOP, hemoglobin remained significant different (between-group difference, 0.25mmol/L [95% CI 0.05 to 0.44], p = 0.014).

Increase in heart rate was significantly lower in the TXA-group versus the placebo-group (-4.6bpm versus 2.5bpm; between-group difference, 7.1bpm [95% CI 1.53 to 12.69], p = 0.013). After correction^a^ for pain and LOP, heart rate remained significant different (between-group difference, 6.9bpm [95% CI 1.19 to 12.52], p = 0.018).

One (2.0%) patient had a minor complication in the TXA-group; ≥1.5 points hemoglobin decrease, versus nine (17.3%) in the placebo-group; hematemesis n=1, melena n=1, infected abdominal wall hematoma n=1, and TXA for ≥1.5points hemoglobin decrease n=7; (RR: 0.12 [95% CI 0.02 to 0.90], p = 0.016). After correcting^b^ for LOP, surgeon and color cartridge, minor complications remained significant different (p = 0.032).

No significant differences were found between surgeons and the use of hemostatic clip devices between both groups. Details are displayed below.

|  | Surgeon (number of patients) | Number of patients in which a clips devices was used | p-value |
| --- | --- | --- | --- |
| TXA | 1 (n = 14) | 9 (64%) | p = 0.299 |
|  | 2 (n = 20) | 16 (80%) |  |
|  | 3 (n = 14) | 9 (64%) |  |
|  | 4 (n = 1) | 0 (0%) |  |
| Placebo | 1 (n = 18) | 14 (78%) | p = 0.810 |
|  | 2 (n = 25) | 21 (84%) |  |
|  | 3 (n = 6) | 5 (83%) |  |
|  | 4 (n = 3) | 3 (100) |  |

Abbreviations; TXA: tranexamic acid; CI: confidence interval; LOP: Length of procedure; mmol/L: millimol per liter; bpm: beats per minut; RR: relative risk.
